# Translation, cultural adaptation, and psychometric validation of the Simplified Chinese Athlete Sleep Behavior Questionnaire (ASBQ-CN) in Chinese athletes

**DOI:** 10.1371/journal.pone.0345641

**Published:** 2026-03-31

**Authors:** Leo Guohui Lin, Zora Yuyang Zhang, Jeremy Rui Chang, Matthew W. Driller, Jiebin Huang, Tony Kwok Wing Lee, Siu-Ngor Fu, Arnold Yu Lok Wong

**Affiliations:** 1 Department of Rehabilitation Sciences, The Hong Kong Polytechnic University, Hong Kong SAR, China; 2 Department of Sports Medicine and Rehabilitation, Beijing Sport University, Beijing, China; 3 Sport, Performance, and Nutrition Research Group, School of Allied Health, Human Services, and Sport, La Trobe University, Melbourne, Australia; 4 Research Institute for Smart Ageing, The Hong Kong Polytechnic University, Hong Kong SAR, China; ISSEP Kef: Universite de Jendouba Institut Superieur du Sport et de l'Education Physique du Kef, TUNISIA

## Abstract

**Objective:**

To translate, adapt, and validate the Simplified Chinese Athlete Sleep Behavior Questionnaire (ASBQ-CN) for both adolescent and adult athletes.

**Design:**

Cross-sectional psychometric evaluation.

**Methods:**

A total of 480 Chinese athletes (19.9 ± 4.4 years) and 408 non-athletes (23.3 ± 3.9 years, for discriminant validity evaluation) were recruited. Psychometric testing included internal consistency, test–retest reliability, content validity, exploratory and confirmatory factor analyses, and convergent and discriminant validity. For robust factor analysis of ordinal data, parallel analysis with polychoric correlations and ordinary least squares estimations were choose.

**Results:**

The ASBQ-CN demonstrated acceptable internal consistency (Cronbach’s α_total_ of 0.76) and test–retest reliability (intraclass correlation coefficient of 0.78). The standard error of measurement and minimal detectable change were 3.13 and 8.67, respectively. Content validity achieving indices of 1.0 at toth item- and scale-level. Factor analyses supported a four-factor structure with acceptable model fit indices (comparative fit index = 0.95, Tucker Lewis index = 0.94, root mean square error of approximation = 0.06, and standardized root mean square residual = 0.09). Convergent validity was confirmed by moderate correlations with the Pittsburgh Sleep Quality Index (r_total_ = 0.44) and Sleep Hygiene Index (r_total_ = 0.50). Although total ASBQ-CN scores did not significantly differ between athletes and non-athletes, significant item-level differences were identified (p < 0.0028). Furthermore, athletes with poor sleep hygiene showed significant higher ASBQ-CN score (p < 0.001).

**Conclusion:**

The ASBQ-CN is a valid and reliable instrument for evaluating sleep behavior in both adolescent and adult Chinese athletes, which could inform personalized interventions.

## Introduction

Optimal sleep is essential for maintaining health and athletic performance. Longitudinal studies suggest that athletes who obtain insufficient sleep (≤8 hours) are at increased risk of infections and musculoskeletal injuries [1]. Optimal sleep duration and quality are associated with improved strength and speed across various sports disciplines [2]. Conversely, sleep deprivation and poor sleep quality impair motor coordination, reaction time, cognitive function, and decision-making, all of which are vital to athletic success [3]. Given the importance of sleep for athletes, the high prevalence of sleep problems in this population is a significant concern.

Sleep disturbances are highly prevalent among athletes. Global epidemiological surveys indicate that 49.0% of adult athletes and 62.5% of adolescent athletes experience sleep-related complaints [4,5]. Similarly, a questionnaire-based survey in China reported that 62.3% of athletes met the criteria for sleep disturbances [6]. These issues range from insufficient and inefficient sleep to sleep disturbances driven by general factors (e.g., stress, anxiety) and sport-specific behaviors (e.g., intensive training schedules, pre-competition pressures). [7] The higher prevalence of sleep complaints in adolescent athletes may also be attributed to differences in sleep-related behaviors. Younger athletes tend to spend extended time in bed and have earlier wake-up times. [8] These behaviors are associated with a higher frequency and longer duration of awakening after sleep [9]. Collectively, the high prevalence of sleep problems and the influence of sleep-related behaviors on sleep underscore the importance of assessing sleep and related behaviors in both adult and adolescent athletes.

Sleep in athletes can be assessed using polysomnography (PSG), actigraphy, validated sleep questionnaires, and sleep diaries [10]. Although PSG is the gold standard for objective sleep assessment, it is resource-intensive and impractical for routine use, especially among elite athletes [10]. Although actigraphy is a wearable technology for long-term monitoring of sleep, its accessibility and cost may limit widespread application [10]. In contrast, validated self-report questionnaires are cost-effective, easy to administer, and more appropriate for large-scale or routine monitoring [10]. Instruments such as the Pittsburgh Sleep Quality Index (PSQI) [11] and Sleep Hygiene Index (SHI) [12] are widely used in the general population. However, these questionnaires do not address the unique sleep challenges faced by athletes. The Athletes Sleep Screen Questionnaire (ASSQ) is a sport-specific instrument for identifying athletes who may need further clinical evaluation and intervention [13]. Nevertheless, the ASSQ does not include assessing modifiable sleep behaviors to guide tailored sleep interventions.

The Athlete Sleep Behavior Questionnaire (ASBQ) is a validated tool specifically developed to evaluate sleep-related behaviors in athletes [14]. It comprises 18 items derived from the SHI [12], the International Classification of Sleep Disorders, and other relevant sources [14]. Intervention strategies tailored to ASBQ profiles have been shown to successfully improve athletes’ sleep outcomes [15].

The ASBQ has been translated and validated in multiple languages, including Turkish [16], Brazilian Portuguese [17], Japanese [18], and Arabic [19]. However, a validated Chinese version is not yet available. Although the ASBQ has been administered to adolescent athletes [20], its psychometric properties in this group remain unestablished. Furthermore, prior studies examined the factor structure using principal component analysis (PCA) [14,16], which is a method appropriate for data reduction but limited in its ability to identify latent constructs, as it does not distinguish between common and unique variance [21]. In contrast, exploratory factor analysis (EFA) models shared variance and thus provide a more robust theoretical framework for uncovering factor structures [21]. To address these gaps, this study aimed to translate, cross-culturally adapt, and evaluate the psychometric properties of the Simplified Chinese version of the Athlete Sleep Behavior Questionnaire (ASBQ-CN) in both adolescent and adult athlete populations in China. Additionally, non-athlete participants were recruited to evaluate the discriminant validity using the known-groups method, comparing groups expected to differ in sleep behaviors.

## Methods

This study was conducted in accordance with the Declaration of Helsinki. Participants were recruited between November 1, 2024, and April 1, 2025. A convenience sample of eligible elite athletes was recruited from the Ersha sports institute in Guangzhou, China, during their winter training (basic preparation) phase to evaluate the reliability and validity of the ASBQ-CN. Where non-athletes were recruited from mainland China (Henan, Guizhou, and Guangdong provinces, via WeChat) and Hong Kong (via the university-wide email distribution list of The Hong Kong Polytechnic University) to evaluate the discriminant validity. All participants, or their legal guardians for adolescent athletes, received detailed study information and provided written informed consent prior to participation, either in person (athletes) or electronically (non-athletes). Ethical approval was obtained from the Institutional Review Board of The Hong Kong Polytechnic University (Ref: HSEARS20241024006). The study followed COSMIN guidelines for evaluating the measurement properties of health-related questionnaires [22]. All questionnaires were administered via a Chinese online platform (https://www.wjx.cn), with all items set as mandatory to minimize missing data.

### Translation and cultural adaptation

The translation and cultural adaptation process followed the guidelines of the Translation and Cultural Adaptation Group of the International Society for Pharmacoeconomics and Outcomes Research (ISPOR) [23]. The full translation and adaptation process is summarized in Table 1:

**Table 1:** The process of translation and cultural adaptation.

Steps	Description
1. Preparation	Permission to translate and validate the ASBQ was obtained from the original developer.
2. Forward translation	Two independent translators, both native Chinese speakers fluent in English, translated the ASBQ into Simplified Chinese. One translator was a sports physiotherapist familiar with the questionnaire’s concepts, while the other was a professional bilingual translator with no prior knowledge of the questionnaire.
3. Reconciliation	The two forward translations were harmonized through consensus discussions involving both translators and the research team.
4. Back-translation	Two native English speakers fluent in Simplified Chinese, and blinded to the original ASBQ, independently back-translated the reconciled version into English.
5. Expert committee review	An expert panel, including the original developer, bilingual researchers, and specialists in sports science and sleep medicine, reviewed all versions. Discrepancies in semantics and cultural relevance were resolved by consensus.
6. Proofreading	Two independent proofreaders reviewed the Simplified Chinese version for spelling, grammar, punctuation, and clarity.
7. Cognitive interviewing	Pilot testing was conducted with 23 athletes in China to examine clarity, comprehensibility, and cultural relevance. Feedback was used to make minor modifications.

### Psychometric testing

All participants completed the ASBQ-CN, the Simplified Chinese version of the Pittsburgh Sleep Quality Index (PSQI-CN), and the Sleep Hygiene Index (SHI-CN). Demographic information (e.g., gender, age) and activity-related variables (e.g., weekly training or exercise frequency, session duration) were collected. To assess test-retest reliability, a randomly selected subset of athletes completed the ASBQ-CN again after a 7-day interval. According to COSMIN guidelines, a sample size of at least 100 participants is considered adequate for evaluating test-retest reliability. To account for potential attrition, 143 athletes from five randomly selected sports were initially invited to the retest assessment. Of these, 111 completed the retest (78% response rate), yielding a final sample size that exceeded the recommended minimum. Before to the second administration, researchers interviewed these athletes regarding their activities during the last week. Those who reported substantial changes (e.g., competition, travel, or cessation of training) were excluded from the test-retest analysis.

#### Participants.

According to COSMIN guidelines, at least seven participants per item are recommended for factor analysis [22], requiring a minimum sample size of 126 for 18-item ASBQ. However, 150 and more participants is generally advised for confirmatory factor analysis (CFA) [21]. To support CFA and known-groups validity analyses, a target sample of 200 athletes and 200 non-athletes was set.

Individuals were recruited if they were [14]: (1) native Mandarin speakers proficient in Simplified Chinese; and (2) either athletes competing at the national or international level, or non-athletes who did not participate in any sports teams and exercised less than four times per week. Exclusion criteria included [14]: (1) being a parent of a child under two years old; or (2) having a diagnosed sleep disorder.

#### Instruments.

**The Athlete Sleep Behavior Questionnaire (ASBQ) [14]:** The ASBQ is an 18-item self-report measure of sleep behaviors over the past month. Items are rated on a 5-point Likert scale (1 = never to 5 = always), with higher scores indicating poorer sleep behavior across three domains: routine/environmental factors, behavioral factors, and sport-related factors.

**The Pittsburgh Sleep Quality Index (PSQI) [11]:** The PSQI is a 19-item questionnaire assessing sleep quality and disturbances across seven domains (e.g., subjective sleep quality, sleep latency), each scored from 0 to 3. Higher global scores (0–21) indicate greater sleep difficulty. The simplified Chinese version of PSQI (PSQI-CN) has demonstrated good reliability and validity in Chinese athletes [24].

**The Sleep Hygiene Index (SHI) [12]:** The SHI contains 13 items evaluating sleep-related behaviors. The validated simplified Chinese version of SHI (SHI-CN) uses dichotomous (yes/no) responses [25]. Total scores range from 0 to 13, with higher scores indicating poorer sleep hygiene. A score ≥ 5.5 indicates poor sleep hygiene.

### Statistical analysis

Statistical analyses were conducted using IBM SPSS Statistics (V.29.0) and R (V 4.2.2). Descriptive statistics were presented as means ± standard deviation (SD) for continuous variables and frequency (percentage) for categorical variables. No missing data was observed due to mandatory online responses.

### Reliability

#### Internal consistency.

Internal consistency was assessed using Cronbach’s alpha (α) and McDonald’s omega (ω). We calculated 95% confidence intervals (CIs) using 10000 bootstrap resamples via the MBESS package in R. Alpha values ≥ 0.7 and omega values ≥ 0.65 were considered acceptable [26]. Item-level internal consistency was further examined using Cronbach’s alpha if item deleted.

#### Test-retest reliability.

A 7-day interval between administrations minimized short-term memory effects and potential behavioral changes over a longer period. Tests were administrated to athletes at the same time of day. Test-retest reliability was evaluated using the intraclass correlation coefficient (ICC 3,1; two-way mixed model, absolute agreement) [27]. ICCs were interpreted as: < 0.5 = poor; 0.5–0.75 = moderate; 0.75–0.9 = good; and > 0.9 = excellent.

#### Measurement errors.

The standard error of measurement (SEM) was calculated as SEM = SD * √(1-ICC), to evaluate the precision of individual test scores [27]. The minimal detectable difference (MDD), representing the smallest change that can be interpreted as a real difference beyond measurement errors, was computed as MDD = 1.96 * √2 * SEM [27].

### Validity

#### Content validity.

Assessed using the Content Validity Index (CVI) [28]. Ten experts in sports and sleep health rated item equivalence of the ASBQ-CN with reference to the original version using a 4-point scale (1 = non-equivalent item; 2 = item needs major revision to assess equivalence; 3 = equivalent item, needs minor changes; 4 = absolutely equivalent). The item-level CVI (I-CVI) was calculated as the proportion of experts’ rating an item as either 3 or 4. Items with I-CVI score < 0.78 should be revised [28]. The average scale level (S-CVI/Ave) was calculated as the mean score of all I-CVI [28]. S-CVI/Ave ≥ 0.9 indicated excellent content validity [28].

#### Construct validity.

The total dataset was randomly split into two equal subsamples using random number generation in Microsoft Excel. The first subsample was used for exploratory factor analysis (EFA) to identify the underlying components of the questionnaire. Subsequently, the second subsample was used for confirmatory factor analyses (CFA) to evaluate the model fit of components structure derived from the EFA. The Kaiser-Meyer-Olkin (KMO) Measure of Sampling Adequacy and Bartlett’s Test of Sphericity were used to assess the feasibility of the data for factor analysis. A KMO value >0.7 and a Bartlett’s test p-value <0.05 were considered suitable for factor analysis [21]. The number of factors in the EFA was determined using parallel analysis based on polychoric correlations and the mean eigenvalue criterion, with principal component analysis (PCA) used as the extraction method [21]. EFA employed ordinary least squares estimations based on polychoric correlations [21]. Oblique (ProMax) rotation was used to allow for potential correlation among factors [21]. Model fit in the CFA was evaluated using the comparative fit index (CFI, > 0.95), Tucker Lewis Index (TLI, > 0.95), root mean square error of approximation (RMSEA, < 0.08), and standardized root mean square residual (SRMR, < 0.08) [29].

#### Discriminant validity.

Discriminant validity was evaluated using known-groups hypothesis testing [22]. First, ASBQ-CN scores were compared between athletes and non-athletes [14]. We hypothesized that athletes would have total scores equal to or higher than those of the general population and would score higher on sports-related items [14,16,19]. Second, ASBQ-CN scores were compared between athletes with good versus poor sleep hygiene status, classified by the SHI-CN cutoff point (≥ 5.5) [25]. We hypothesized that athletes with poor sleep hygiene would have significantly higher scores, supporting the instruments discriminate validity. For normally distributed data, independent samples t-tests were applied; for non-normally distributed data, Mann-Whitney U test were used. Analyses were conducted at both the item and global score levels, consistent with the original version [14]. Statistical significance was set at p < 0.05 for total scores, with Bonferroni correction applied to item-level comparisons (p < 0.0028, 0.05/18 items).

#### Convergent validity.

This was evaluated using Spearman’s rank correlation between ASBQ-CN scores and those from validated instruments, including the PSQI-CN [24] and SHI-CN [25]. The strength of the correlations was interpreted as follows: 0.00–0.10 = negligible; 0.10–0.39 = weak; 0.40–0.69 = moderate; 0.7–0.89 = strong; and 0.9–1.0 = very strong [30].

## Results

### Translation and cultural adaptation

During the forward translation process, minor modifications were made to enhance cultural relevance. Specifically, references to “roommates” were added in Items 13 and 14, and the term “dorm” was included in Item 16 to better reflect the typical living arrangements of Chinese professional athletes, who often reside in shared dormitories at sports institutes. These adaptations were approved by the original developer, the research team, and the second forward translator. No major discrepancies were identified during this process.

An expert committee, in consultation with the original questionnaire developer, evaluated the equivalence of each item and response option using a 4-point scale. No items scored below 3; items rated as “3” (minor revision required) were modified accordingly, resulting in the prefinal version of the ASBQ-CN.

Pilot testing: The prefinal ASBQ-CN was pilot tested with 23 judo athletes (11 females and 12 males; mean age 21.6 years, range 17–29 years). All participants reported that the items and response options were clear and easy to understand during follow-up interviews.

### Psychometric testing

#### Participants characteristics.

**Fig 1:**
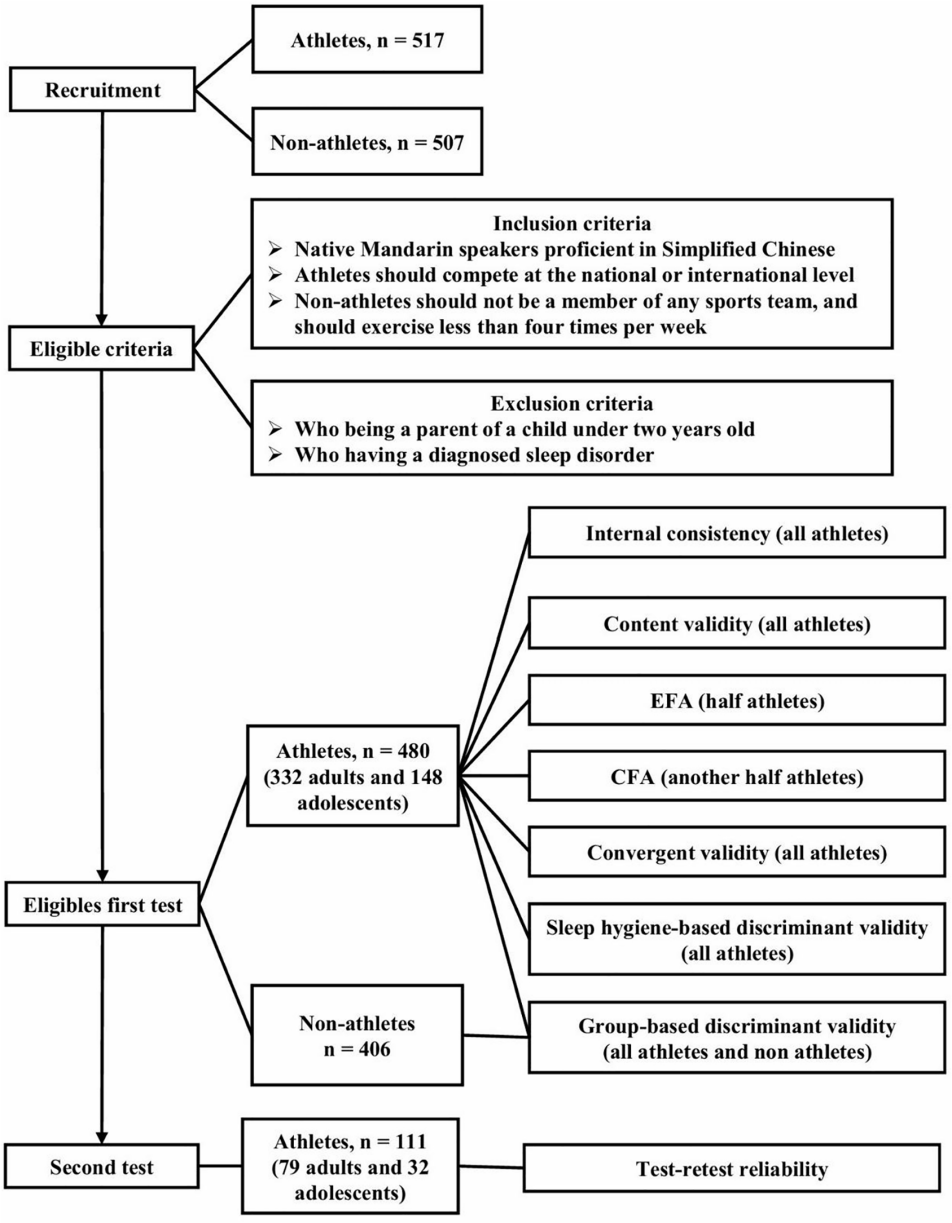
Study flow of psychometric testing.

Of the 517 athletes and 507 non-athletes who responded to recruitment, the final eligible sample of psychometric testing comprised 480 athletes and 406 non-athletes. The athlete cohort included 332 adults (aged ≥ 18 years) and 148 adolescents (aged < 18 years). The majority of participants fell within the translational age ranges: 178 (53.6%) adult athletes were aged 18–21 years, and 105 adolescent athletes (71%) were aged 15–17 years. Most participants competed at the provincial level in national competitions. Athletic classifications ranged from the National Second Class to Master Class, with approximately 9% of the athletes having represented China in international competition. The participants recruitment and assessment process are presented in Fig 1. The recruited athletes participated in artistic swimming (n = 16), badminton (n = 31), basketball (n = 20), diving (n = 26), fencing (n = 31), gymnastics (n = 36), swimming (n = 37), table tennis (n = 28), tennis (n = 16), track and field (n = 56), trampoline (n = 28), volleyball (n = 39), water polo (n = 31), weightlifting (n = 42), and Wushu (n = 33). The demographic characteristics of the participants are summarized in Table 2.

**Table 2:** Descriptive characteristics of participants.

Groups		Test		Retest	
		Number (Female/Male)	Ages (years)	Number (Female/Male)	Ages (years)
Athletes	Total	480 (226/254)	19.9 ± 4.4	111 (67/44)	19.6 ± 3.8
	Adult (≥ 18y)	332 (129/203)	22.0 ± 3.4	79 (43/36)	21.3 ± 2.8
	Adolescent (<18y)	148 (97/51)	15.2 ± 1.9	32 (24/8)	15.3 ± 1.9
Non-Athletes		406 (243/163)	23.3 ± 3.9	NA	NA

NA = not applicable.

#### Reliability.

The ASBQ-CN demonstrated good internal consistency. For the total sample, Cronbach’s α was 0.75 (95% CI 0.72 to 0.78) and McDonald’s ω was 0.76 (95% CI 0.73 to 0.79). For adult athletes, Cronbach’s α was 0.74 (95% CI 0.69 to 0.78) and McDonald’s ω was 0.74 (95% CI 0.67 to 0.78). For adolescent athletes, Cronbach’s α was 0.78 (95% CI 0.72 to 0.83) and McDonald’s ω was 0.79 (95% CI 0.74 to 0.84). Item-deleted Cronbach’s α values ranged from 0.73 to 0.77 for the total sample, 0.71 to 0.76 for adult athletes, and 0.75 to 0.80 for adolescent athletes.

Test-retest reliability was acceptable across all groups. The ICC (95% CI) was 0.78 (0.69–0.84) for the total sample, 0.78 (0.66–0.85) for adult athletes, and 0.73 (0.52–0.86) for adolescent athletes. The SEM were 3.13, 3.31, and 3.05, respectively, and the MDC was 8.67, 9.18, and 8.45.

#### Validity.

Content validity was confirmed by the expert committee and the original questionnaire developer. All items demonstrated excellent equivalence, with I-CVI of 1.0. The S-CVI also showed excellent content validity (S-CVI/Ave = 1.0).

All athletes were randomly and equally divided into two groups for EFA and CFA. The EFA subsample (n = 240) demonstrated adequate sampling adequacy (KMO = 0.71) and a significant Bartlett’s test of sphericity (chi-square = 783.11, p < 0.01). Parallel analysis suggested a 4-factor structure, with the extracted components presented in Table 3.

**Table 3:** EFA factor structure of the Simplified Chinese Versions of the Athlete Sleep Behavior Questionnaire (ASBQ-CN).

Factors	Factor 1	Factor 2	Factor 3	Factor 4
F 1 Training/competition schedule				
ASBQ-CN # 3	0.47			
ASBQ-CN # 11	0.49			
ASBQ-CN # 15	0.28			
ASBQ-CN # 17	0.81			
ASBQ-CN # 18	0.84			
F 2 Stress and recovery napping				
ASBQ-CN # 1		−0.28		
ASBQ-CN # 7		0.32		
ASBQ-CN # 9		0.91		
ASBQ-CN # 10		0.75		
F 3 Sleep-disturbing stimuli				
ASBQ-CN # 2			0.48	
ASBQ-CN # 4			0.8	
ASBQ-CN # 5			0.46	
ASBQ-CN # 6			0.42	
ASBQ-CN # 8			0.56	
F 4 Sleep disturbances/environment				
ASBQ-CN # 12				0.49
ASBQ-CN # 13				0.77
ASBQ-CN # 14				0.57
ASBQ-CN # 16				0.33

The CFA subsample (n = 240) also showed adequate sampling adequacy (KMO = 0.79) and a significant Bartlett’s test (chi-square = 772.48, p < 0.01). The initial CFA indicated an acceptable model fit: CFI = 0.95, TLI = 0.94, RMSEA = 0.06 (90% CI 0.05–0.08), and SRMR = 0.09. After removing ASBQ item 1, the CFA model fit further improved: CFI = 0.96, TLI = 0.95, RMSEA = 0.06 (90% CI 0.05–0.08), and SRMR = 0.08. Detailed parameters estimates are presented in Table 4. Inter-factor correlation ranged from 0.60 to 0.82 (Table 5).

**Table 4:** CFA factor structure of the Simplified Chinese Versions of the Athlete Sleep Behavior Questionnaire (ASBQ-CN).

Factor	Item	All items			After remove ASBQ_1		
		Loding	P	Residual	Loding	P	Residual
1	ASBQ-CN # 3	0.33	< 0.01	0.89	0.38	< 0.01	0.86
	ASBQ-CN # 11	0.49	< 0.01	0.76	0.56	< 0.01	0.69
	ASBQ-CN # 15	0.52	< 0.01	0.73	0.63	< 0.01	0.60
	ASBQ-CN # 17	0.68	< 0.01	0.53	0.68	< 0.01	0.54
	ASBQ-CN # 18	0.54	< 0.01	0.70	0.54	< 0.01	0.71
2	ASBQ-CN # 1	− 0.04	0.55	0.998	NA	NA	NA
	ASBQ-CN # 7	0.66	< 0.01	0.57	0.73	< 0.01	0.47
	ASBQ-CN # 9	0.79	< 0.01	0.38	0.72	< 0.01	0.48
	ASBQ-CN # 10	0.81	< 0.01	0.34	0.80	< 0.01	0.36
3	ASBQ-CN # 2	0.42	< 0.01	0.82	0.38	< 0.01	0.85
	ASBQ-CN # 4	0.47	< 0.01	0.78	0.42	< 0.01	0.82
	ASBQ-CN # 5	0.53	< 0.01	0.72	0.66	< 0.01	0.56
	ASBQ-CN # 6	0.54	< 0.01	0.71	0.56	< 0.01	0.68
	ASBQ-CN # 8	0.46	< 0.01	0.79	0.44	< 0.01	0.80
4	ASBQ-CN # 12	0.37	< 0.01	0.86	0.35	< 0.01	0.87
	ASBQ-CN # 13	0.24	< 0.01	0.94	0.39	< 0.01	0.85
	ASBQ-CN # 14	0.63	< 0.01	0.60	0.68	< 0.01	0.53
	ASBQ-CN # 16	0.60	< 0.01	0.65	0.64	< 0.01	0.59
Model fit							
CFI		0.950			0.959		
TLI		0.941			0.950		
RMSEA		0.063 (90% CI 0.052–0.075)			0.063 (90% CI 0.050–0.075)		
SRMR		0.088			0.082		

**Table 5:** Correlation between factors of CFA factor structure.

Factor-pair	All items	After remove ASBQ_1
**Factor 1 – Factor 2**	0.67	0.64
**Factor 1 – Factor 3**	0.81	0.82
**Factor 1 – Factor 4**	0.81	0.62
**Factor 2 – Factor 3**	0.79	0.79
**Factor 2 – Factor 4**	0.84	0.71
**Factor 3 – Factor 4**	0.83	0.60

**Fig 2:**
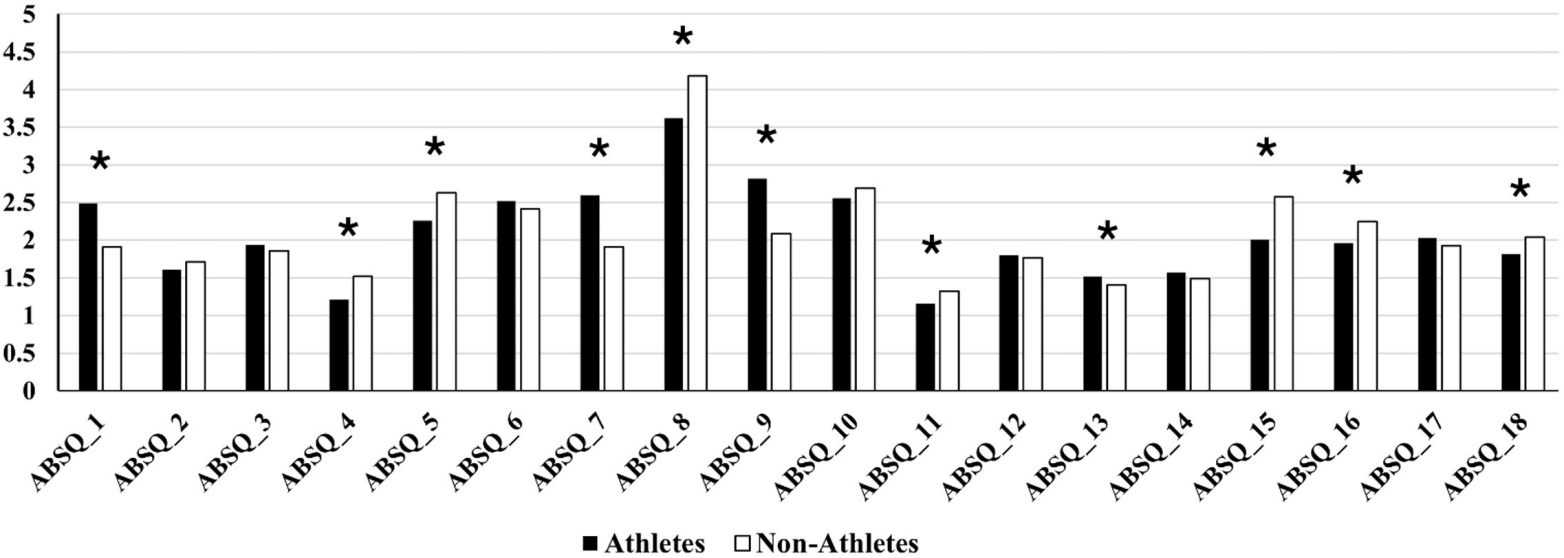
Mean differences in item scores between athletes and non-athletes. ASBQ = Simplified Chinese version of the Athlete Sleep Behavior Questionnaire. * = significant difference (p < 0.0028).

For the known-group validity analyses, total ASBQ-CN scores did not significantly differ between athletes and non-athletes (37.5 ± 7.32 vs 37.71 ± 8.89, t = − 0.39, p = 0.70). The mean difference was −0.21 points (95% CI −1.30 to 0.87), representing a negligible effect size (Cohen’s d −0.03, 95% CI −0.16 to 0.11). However, 11 out of 18 item scores showed significant differences (Fig 2). Descriptive statistics for the PSQI-CN, SHI-CN, and ASBQ-CN (total scores) are presented in Table 6.

**Table 6:** The descriptive statistics of the scores of the Simplified Chinese version of the Pittsburgh Sleep Quality Index (PSQI-CN), Sleep Hygiene Index (SHI-CN), and Athlete Sleep Behavior Questionnaire (ASBQ-CN).

Groups		Test			Retest		
		PSQI-CN	SHI-CN	ASBQ-CN	PSQI-CN	SHI-CN	ASBQ-CN
Athletes	Total	6.99 ± 3.34	3.96 ± 2.15	37.50 ± 7.32	7.23 ± 2.99	3.85 ± 2.09	37.41 ± 6.91
	Adult	7.28 ± 3.47	3.94 ± 2.12	38.25 ± 7.21	7.84 ± 3.02	3.86 ± 2.02	38.49 ± 6.69
	Adolescent	6.32 ± 2.94	4.01 ± 2.22	35.81 ± 7.29	6.63 ± 2.85	3.81 ± 2.26	34.75 ± 6.83
Non-Athletes		7.43 ± 3.46	5.52 ± 2.64	37.71 ± 8.89	NA	NA	NA

NA = not applicable.

For behavior-based known-group validity, athletes with poor sleep hygiene demonstrated significantly higher ASBQ-CN scores than those with good sleep hygiene (42.23 ± 6.11 vs 36.2 ± 7.09; Mann-Whitney U = 9657.5, Z = 7.83, p < 0.01). The mean difference was 6.03 points (95% CI 4.64 to 7.42), exceeding 1 SEM. This pattern evident in subgroup analyses. Among adult athletes, ASBQ-CN scores were significantly higher in the poor sleep hygiene group than in the good sleep hygiene group (42.56 ± 5.511 vs 37.18 ± 7.191; t = 6.65, p < 0.001) with a large effect size (Cohen’s d = 0.78, 95% CI 0.5 to 1.1). The corresponding mean difference was 5.38 points (95% CI 3.78 to 6.98), exceeding 2 SEM. A similar difference was observed in adolescent athletes (41.65 ± 7.09 vs 33.86 ± 6.27; Mann-Whitney U = 751.5, Z = 5.78, p < 0.01), with a mean difference of 7.79 points (95% CI 5.16 to 10.41), which exceeded 2 SEM.

The ASBQ-CN demonstrated moderate correlations with related sleep measures, with correlations coefficients ranging from 0.41 to 0.54, all meeting the predefined criteria for moderate associations (r = 0.40–0.60). In the total athlete sample, ASBQ-CN scores were moderately related to both the PSQI-CN (r = 0.44, p < 0.01) and the SHI-CN scores (r = 0.50, p < 0.01). Similar patterns were observed among adult athletes (PSQI-CN: r = 0.43, p < 0.01; SHI-CN: r = 0.49, p < 0.001) and adolescent athletes (PSQI-CN: r = 0.41, p < 0.001; SHI-CN r = 0.54, p < 0.001). Collectively, these findings support the convergent validity of the ASBQ-CN.

## Discussion

This study successfully translated and cross-culturally adapted the ASBQ into simplified Chinese (S1 File). Content validity testing confirmed that the ASBQ-CN is conceptually equivalent to the original English version. The translated questionnaire demonstrated good psychometric properties when tested among Chinese athletes (Table 7). These findings support the use of the ASBQ-CN as a reliable and valid too for assessing sleep behaviors in Chinese athletes and guiding targeted interventions to improve their sleep. However, ASBQ cannot distinguish between athletes and non-athletes.

**Table 7:** Summary of the reliability and validity of the original and various translated version of the Athlete Sleep Behavior Questionnaires.

Version	Validity			Reliability	
	Content	Factor structure (explained variance, %)	Convergent	Internal consistency (if 1 item was deleted)	Test retest
Arabic [19]	NA	CFA: 3 factors (NA); Routine/Environmental factors (Q1, Q5, Q15 - Q18), Behavioral factors (Q2, Q4, Q8, Q10 - Q13) and Sport-related factors (Q3, Q6, Q7, Q9, Q14)	ISI (r = 0.5)	Cronbach’s α = 0.72 (0.70–0.73) McDonald’s ω = 0.725	ICC = 0.88
Brazilian Portuguese [17]	Cvci = 0.89–1.0 Cvct = 0.96	NA	NA	Cronbach’s α = 0.78 (NA)	ICC (95% CI) = 0.857 (0.73–0.93)
Chinese (Simplified)	I-CVI = 1.0 S-CVI/Ave = 1.0	Total: EFA and CFA: 4 factors (NA); Training/competition schedule (Q3, Q11, Q15, Q17, Q18), Stress and recovery napping (Q1, Q7, Q9, Q10), Sleep-disturbing stimuli (Q2, Q4, Q5, Q6, Q8), Sleep disturbances/environment (Q12, Q13, Q14, Q16)	Total: PSQI-CN (r = 0.44); SHI-CN (r = 0.50)	Cronbach’s α = 0.76 (0.73–0.77) McDonald’s ω = 762	ICC (95% CI) = 0.78 (0.69–0.84)
		Adult (NA)	Adult: PSQI-CN (r = 0.43); SHI-CN (r = 0.49)	Cronbach’s α = 0.74 (0.71–0.76)	ICC (95% CI) = 0.78 (0.66–0.85)
		Adolescent (NA)	Adolescent: PSQI-CN (r = 0.41); SHI-CN (r = 0.54)	Cronbach’s α = 0.79 (0.75–0.80)	ICC (95% CI) = 0.73 (0.52–0.86)
English [14]	NA	PCA: 3 factors (69.6%); Routine/Environmental factors (Q1, Q5, Q15 - Q18), Behavioral factors (Q2, Q4, Q8, Q10 - Q13) and Sport-related factors (Q3, Q6, Q7, Q9, Q14)	PSQI (r = 0.38); SHI (r = 0.69)	Cronbach’s α = 0.63 (NA)	ICC (90% CI) = 0.87 (0.80–0.92)
Japanese [18]	NA	NA	NA	Cronbach’s α = 0.62 (NA)	ICC (95% CI) = 0.78 (0.64–0.86)
Korean [31]	NA	NA	Original ASBQ (r = 0.80)	Cronbach’s α = 0.87	ICC (90% CI) = 0.78 (0.62–0.88)
Turkish [16]	NA	PCA: 4 factors (NA); Sport-related factors (Q2, Q3, Q9, Q10, Q16), Sleep quality factors (Q1, Q7, Q8, Q12, Q17), Habitual sleep efficiency factors (Q4, Q5, Q6, Q15), Sleep disturbance factors (Q11, Q13, Q14)	NA	Cronbach’s α = 0.62 (NA)	ICC = 0.85

CFA = Confirmatory factor analysis; EFA = Exploratory factor analysis; ISI = Insomnia Severity Index; ICC = Intraclass correlation coefficient; Cvci = Item level content validity coefficient; Cvct = Total content validity coefficient; NA = not applicable; I-CVI = Item-level content validity index; S-CVI/Ave = Scale level content validity index calculated by average of the I-CVI; PSQI-CN = Chinese version of Pittsburgh Sleep Quality Index; SHI-CN = Chinese version of Sleep Hygiene Index; PCA = Principal component analysis; PSQI = Pittsburgh Sleep Quality Index; SHI = Sleep Hygiene Index; ASBQ = Athlete Sleep Behavior Questionnaire; Q1 = I take afternoon naps lasting two or more hours; Q2 = I use stimulants when I train/compete (e.g., caffeine); Q3 = I exercise (train or compete) late at night (after 7 pm); Q4 = I consume alcohol within 4 hours of going to bed; Q5 = I go to bed at different times each night (more than ±1 hour variation); Q6 = I go to bed feeling thirsty; Q7 = I go to bed with sore muscles; Q8 = I use light-emitting technology in the hour leading up to bedtime (e.g., laptop, phone, television, video games); Q9 = I think, plan and worry about my sporting performance when I am in bed; Q10 = I think, plan and worry about issues not related to my sport when I am in bed; Q11 = I use sleeping pills/tablets to help me sleep; Q12 = I wake to go to the bathroom more than once per night; Q13 = I wake myself and/or my roommate/bed partner with my snoring; Q14 = I wake myself and/or my roommate/bed partner with my muscle twitching; Q15 = I get up at different times each morning (more than ±1 hour variation); Q16 = At home/dormitory, I sleep in a less than ideal environment (e.g., too light, too noisy, uncomfortable bed/pillow, too hot/cold); Q17 = I sleep in foreign environments (e.g., hotel rooms); Q18 = Travel gets in the way of building a consistent sleep-wake routine.

The internal consistency of the ASBQ-CN was very good. Both Cronbach’s α and McDonald’s ω coefficients exceeding recommended thresholds. The Cronbach’s α values were higher than those reported in the Japanese [18], Turkish [16], and original English [14] versions, and were comparable to those of the Arabic [19] and Brazilian Portuguese [17] versions. Among previous validation studies, only the Arabic version reported McDonald’s ω value. In the present study, ω values were slightly higher than that reported for the Arabic version. These results indicating equal or superior internal consistency of the ASBQ-CN.

The test-retest reliability of the ASBQ-CN was lower than that reported for the English [14], Arabic [19], Turkish [16], and Brazilian Portuguese [17] versions, but remained acceptable across all groups. Notably, these values were comparable to those observed in the Japanese version [18]. These metrics indicate that the ASBQ-CN items consistently measure sleep behavior and provide reliable scores across repeated assessments.

Among all translated versions, only the Brazilian Portuguese version and the ASBQ-CN evaluated the content validity [17]. The ASBQ-CN demonstrated a higher content validity at both item and the scale levels [17], confirming the equivalence between the ASBQ-CN and the original English version [14].

Construct validity of the ASBQ-CN was supported by factor analysis. EFA revealed a four-factor structures: training/competition schedule, stress and recovery napping, sleep-disturbing stimuli, and sleep disturbances/environment (Table 7). Prior studies on the ASBQ, including the Arabic, English, and Turkish versions [14,16,19], have reported varying factor structures. The English and Turkish versions employed PCA to examine the factor structure [14, 16], whereas the Arabic version adopted the factor structure proposed in the original English model [19]. However, PCA is primarily a data reduction technique that maximizes total variance (common, unique, and error variance) and is therefore limited in its ability to capture latent constructs [21]. In contrast, the present study applied EFA, which models shared variance among items and is considered a more theoretically appropriate approach for identifying latent factors in psychometric instruments [21]. The four-factor structure identified in our study differed from those reported in the Arabic, English, and Turkish versions (Table 7) [14,16,19]. These differences may be attributable to methodological differences, the unique training schedules of our athletic cohort (e.g., early morning training), and the inclusion of adolescent athletes. Although item 15 of the ASBQ-CN showed a relatively low loading in the EFA, it was retained due to its theoretical relevance to sleep-related behaviors in athletes, [14,19] and its contribution to overall internal consistency.

Notably, ASBQ-CN item 1 (napping >2 hours) demonstrated a negative loading on certain factors during EFA. This finding suggests that, in this population, longer naps may serve a stress-reducing rather than stress-inducing function. Given the typical early morning (~7:00) and afternoon (~15:00) training schedules in our sample athletes, this finding aligns with previous research [32]. This phenomenon indicating that extended napping before 16:00 does not impair nighttime sleep and may be an important recovery strategy for athletes managing physical and psychological stress. Therefore, further refinement of item 1 – particularly to clarify its intent and ensure alignment with athletes’ typical routines – may be warranted to improve the scale’s internal consistency and culture relevance.

The CFA results further confirmed the construct validity of the four-factor structure identified by the EFA. The initial CFA further confirmed the possible miss fitted issue of item ASBQ-CN item 1. This item showed a very low and non-significant factor loading (lambda = − 0.04, p = 0.55), with residual variance close to unity. Based on converging evidence from both the EFA and CFA, item 1 was removed and the CFA was re-estimated. The revised model demonstrated improved overall fit and stable, statistically significant factor loading for all remaining items. The fit indices of both CFA models exceeded those reported for Arabic version (CFI = 0.70, TLI = 0.65, RMSEA = 0.07, and SRMR = 0.07) [19]. These results further supported the structural robustness of the ASBQ-CN.

Known-group validity across athletes and non-athletes was partially supported. No significant differences in total ASBQ-CN scores were found between athletes and non-athletes, consistent with findings from the Arabic version [19]. However, item-level comparisons revealed significant differences: athletes scored higher on sport-specific items (e.g., Q7 and Q9), while non-athletes demonstrated poorer general sleep hygiene (e.g., Q4). These patterns concur with previous observations in the English, Arabic, and Turkish versions [14,16,19]. Although PSQI-CN scores did not differ significantly between groups, the general population exhibited significantly poorer sleep hygiene, as indicated by higher SHI-CN scores. Conversely, ASBQ-CN scores revealed similar level of bad sleep behaviors in both athletes and the general population. This suggests that the ASBQ may be more sensitive to detecting athlete-specific sleep behavior patterns than general sleep behaviors tools such as the SHI. This could be attributed to the inclusion of sport-specific items in the ASBQ, as revealed in our item-level analysis.

This study is the first to examine known-group validity based on sleep hygiene status. The ASBQ-CN effectively differentiated between athletes with good and poor sleep hygiene defined by SHI-CN [25], with the difference exceeding 1 SEM. Subgroup analyses further confirmed this trend in both adults and adolescents, indicating the ASBQ-CN’s sensitivity to behavioral differences across age groups and supporting its discriminant validity.

Convergent validity was supported by moderate correlations between the ASBQ-CN with the PSQI-CN and SHI-CN, with stronger associations observed for the SHI, consistent with findings from the original ASBQ validation [14]. These correlations were stable across adult and adolescent subgroups (Table 7). The result suggest that the ASBQ-CN effectively captures factors that impair sleep quality and is more strongly associated with sleep hygiene behaviors, further supporting its construct validity.

### Strengths and limitations

This study is the first to validate the ASBQ-CN in both adult and adolescent Chinese athletes, adhering to COSMIN guidelines [22]. Construct validity was rigorously evaluated using factor analysis based on classical test theory.

However, several limitations should be noted. First, convergent validity was only assessed using subjective measures (PSQI and SHI). Future studies should incorporate objective tools such as actigraphy or polysomnography to further validate their relationship with the ASBQ-CN. Second, data were collected during the regular training phase, which may not reflect sleep behaviors across different training and competition seasons. Future research should explore seasonal variations in sleep patterns.

## Conclusion

This study demonstrates that the ASBQ-CN is a reliable and valid tool for assessing sleep behavior among Chinese athletes. Coaches and researchers may use the ASBQ-CN to routinely identify and address maladaptive sleep behaviors, ultimately aiming to improve athletes’ sleep quality and recovery. Further studies are warranted to gain detailed insights into sport-specific sleep behaviors.

## Supporting information

S1 File The Simplified Chinese version of the Athlete Sleep Behavior Questionnaire (ASBQ-CN). (DOCX)
