# Supplementary material for: Translation, cultural adaptation, and psychometric validation of the Simplified Chinese Athlete Sleep Behavior Questionnaire (ASBQ-CN) in Chinese athletes
Source: PLoS One. 2026 Mar 31;21(3):e0345641. doi: 10.1371/journal.pone.0345641 (PMC13038020; doi:10.1371/journal.pone.0345641)
Supplement: S1 File — (DOCX) [file pone.0345641.s001.docx]

**Supporting information**

**The Simplified Chinese version of the Athlete Sleep Behavior Questionnaire (ASBQ-CN)**

**Chinese version**

运动员睡眠行为问卷-简体中文版（ASBQ-CN）

| 序号 | 最近一段时间（过去一个月内）…… | 从不 | 很少 | 有时 | 经常 | 总是 |
| --- | --- | --- | --- | --- | --- | --- |
| 1 | 我午睡两小时或更长时间 |  |  |  |  |  |
| 2 | 我在训练或比赛时服用提神物（例如咖啡因） |  |  |  |  |  |
| 3 | 我在晚上7点之后运动（训练或比赛） |  |  |  |  |  |
| 4 | 我在睡前4小时内喝酒 |  |  |  |  |  |
| 5 | 我每晚上床睡觉的时间都不一样（相差超过1小时） |  |  |  |  |  |
| 6 | 我上床睡觉时感到口渴 |  |  |  |  |  |
| 7 | 我上床睡觉时肌肉酸痛 |  |  |  |  |  |
| 8 | 我在睡前1小时内使用发光电子设备（例如笔记本电脑、手机、电视、电子游戏等） |  |  |  |  |  |
| 9 | 当我在床上时，我会思考、计划和担心我的运动表现 |  |  |  |  |  |
| 10 | 当我在床上时，我会思考、计划和担心与我的运动无关的问题 |  |  |  |  |  |
| 11 | 我服用安眠药来帮助入睡 |  |  |  |  |  |
| 12 | 我每晚醒来上厕所超过一次 |  |  |  |  |  |
| 13 | 我因为打鼾而吵醒自己和/或伴侣/室友 |  |  |  |  |  |
| 14 | 我因为肌肉抽搐而惊醒自己和/或伴侣/室友 |  |  |  |  |  |
| 15 | 我每天早上起床的时间都不一样（相差超过1小时） |  |  |  |  |  |
| 16 | 在家里/宿舍时，我的睡觉环境不够理想（例如光线太亮、太吵、床或枕头不舒服、太热或太冷等） |  |  |  |  |  |
| 17 | 我曾在陌生环境中睡觉（例如酒店房间） |  |  |  |  |  |
| 18 | 外出训练/比赛阻碍了我建立稳定的睡眠作息规律 |  |  |  |  |  |

得分：

从不 = 1，很少 =2，有时 = 3，经常 = 4， 总是 = 5 总分：

**English translation**

The Simplified Chinese version of the Athlete Sleep Behavior Questionnaire (ASBQ-CN)

| No. | In recent times (over the last month) … | Never | Rarely | Sometimes | Frequently | Always |
| --- | --- | --- | --- | --- | --- | --- |
| 1 | I take afternoon naps lasting two or more hours |  |  |  |  |  |
| 2 | I use stimulants when I train/compete (e.g. caffeine) |  |  |  |  |  |
| 3 | I exercise (train or compete) late at night (after 7pm) |  |  |  |  |  |
| 4 | I consume alcohol within 4 hours of going to bed |  |  |  |  |  |
| 5 | I go to bed at different times each night  (more than ±1 hour variation) |  |  |  |  |  |
| 6 | I go to bed feeling thirsty |  |  |  |  |  |
| 7 | I go to bed with sore muscles |  |  |  |  |  |
| 8 | I use light-emitting technology in the hour leading up to bedtime (e.g. laptop, phone, television, video games) |  |  |  |  |  |
| 9 | I think, plan and worry about my sporting performance when I am in bed |  |  |  |  |  |
| 10 | I think, plan and worry about issues not related to my sport when I am in bed |  |  |  |  |  |
| 11 | I use sleeping pills/tablets to help me sleep |  |  |  |  |  |
| 12 | I wake to go to the bathroom more than once per night |  |  |  |  |  |
| 13 | I wake myself and/or my bed partner/roommates with my snoring |  |  |  |  |  |
| 14 | I wake myself and/or my bed partner/roommates with my muscle twitching |  |  |  |  |  |
| 15 | I get up at different times each morning  (more than ±1 hour variation) |  |  |  |  |  |
| 16 | At home/dorm, I sleep in a less than ideal environment (e.g. too light, too noisy, uncomfortable bed/pillow, too hot/cold) |  |  |  |  |  |
| 17 | I sleep in foreign environments (e.g. hotel rooms) |  |  |  |  |  |
| 18 | Travel gets in the way of building a consistent sleep-wake routine |  |  |  |  |  |

Scoring:

Never = 1, Rarely = 2, Sometimes = 3, Frequently = 4, Always = 5 Total Global Score: _________
